# Effects and potential mechanisms of exercise and physical activity on eye health and ocular diseases

**DOI:** 10.3389/fmed.2024.1353624

**Published:** 2024-03-22

**Authors:** Qiuxiang Zhang, Yuxian Jiang, Chaohua Deng, Junming Wang

**Affiliations:** Department of Ophthalmology, Tongji Hospital, Tongji Medical College, Huazhong University of Science and Technology, Wuhan, China

**Keywords:** exercise, physical activity, ocular diseases, oxidative stress, inflammation

## Abstract

In the field of eye health, the profound impact of exercise and physical activity on various ocular diseases has become a focal point of attention. This review summarizes and elucidates the positive effects of exercise and physical activities on common ocular diseases, including dry eye disease (DED), cataracts, myopia, glaucoma, diabetic retinopathy (DR), and age-related macular degeneration (AMD). It also catalogues and offers exercise recommendations based on the varying impacts that different types and intensities of physical activities may have on specific eye conditions. Beyond correlations, this review also compiles potential mechanisms through which exercise and physical activity beneficially affect eye health. From mitigating ocular oxidative stress and inflammatory responses, reducing intraocular pressure, enhancing mitochondrial function, to promoting ocular blood circulation and the release of protective factors, the complex biological effects triggered by exercise and physical activities reveal their substantial potential in preventing and even assisting in the treatment of ocular diseases. This review aims not only to foster awareness and appreciation for how exercise and physical activity can improve eye health but also to serve as a catalyst for further exploration into the specific mechanisms and key targets through which exercise impacts ocular health. Such inquiries are crucial for advancing innovative strategies for the treatment of eye diseases, thereby holding significant implications for the development of new therapeutic approaches.

## 1 Introduction

Eye health is vital for maintaining physical and mental well-being, which has extensive implications on individual quality of life, economic prosperity of nations, and sustainable development of societies (1). However, global eye health statistics show an alarming picture, with more than 2.2 billion people worldwide suffering from visual impairment, of whom more than 1 billion face moderates to severe visual impairment or even complete blindness (2). Unfortunately, numerous instances of visual impairment within this demographic can be prevented or intervened early, such as cataracts, refractive errors and glaucoma (2). Nevertheless, countless individuals and families endure the repercussions of visual impairment or complete loss of vision due to obstacles in obtaining affordable and high-quality eye care and treatment (2). Recognizing that eye health is a global public priority and repositioning it as a development and health issue, the Global Commission on Eye Health has gained increased attention (1).

In recent years, exercise and physical activity have gained growing attention as cost-effective, feasible and accessible lifestyle interventions for the treatment of various human ailments, notably cardiovascular and other chronic diseases (3). Several studies have indicated that long-term regular exercise and physical activity serve as crucial and modifiable interventions for enhancing the management of diverse medical conditions, including, but not limited to, hypertension, diabetes, obesity, cancer, osteoporosis, osteoarthritis, and depression (4). A cohort study of 110,482 healthy middle-aged adults over an 8 years period revealed that low physical fitness was linked to a higher incidence of mortality from various causes (5). It has been reported that physical inactivity has the highest prevalence among risk factors that can be intervened upon by humans (3). Despite the extensively documented benefits of physical activity in many systemic disorders, exercise is underutilized as an intervention in practice. Astonishingly, only about 26% of men and 19% of women are estimated to meet international physical activity guidelines (6, 7).

The positive impacts of exercise on eye health are increasingly recognized and widely validated by a growing body of research (8–11). This review synthesizes existing studies to describe the potential benefits of exercise and physical activities on common ocular diseases. Based on current epidemiological evidence and exercise guidelines, this review provides exercise recommendations for early to mid-stage patients of specific ocular diseases or high-risk individuals who are physically fit for exercise. It is hoped that this will elevate the awareness and importance of exercise in eye health among various sectors of society. Additionally, this review summarizes and elucidates possible mechanisms through which exercise and physical activity impact common ocular diseases, including direct mechanisms such as lowering intraocular pressure and blood glucose, as well as indirect mechanisms like inhibiting oxidative stress and inflammatory responses, and promoting the release of protective factors. The discussion on mechanisms aims to encourage researchers to delve deeper into unveiling the specific mechanisms and key targets through which exercise positively affects eye health, thereby providing theoretical support for the development of new treatment strategies for ocular diseases.

## 2 Exercise and physical activity

**Figure 1:**
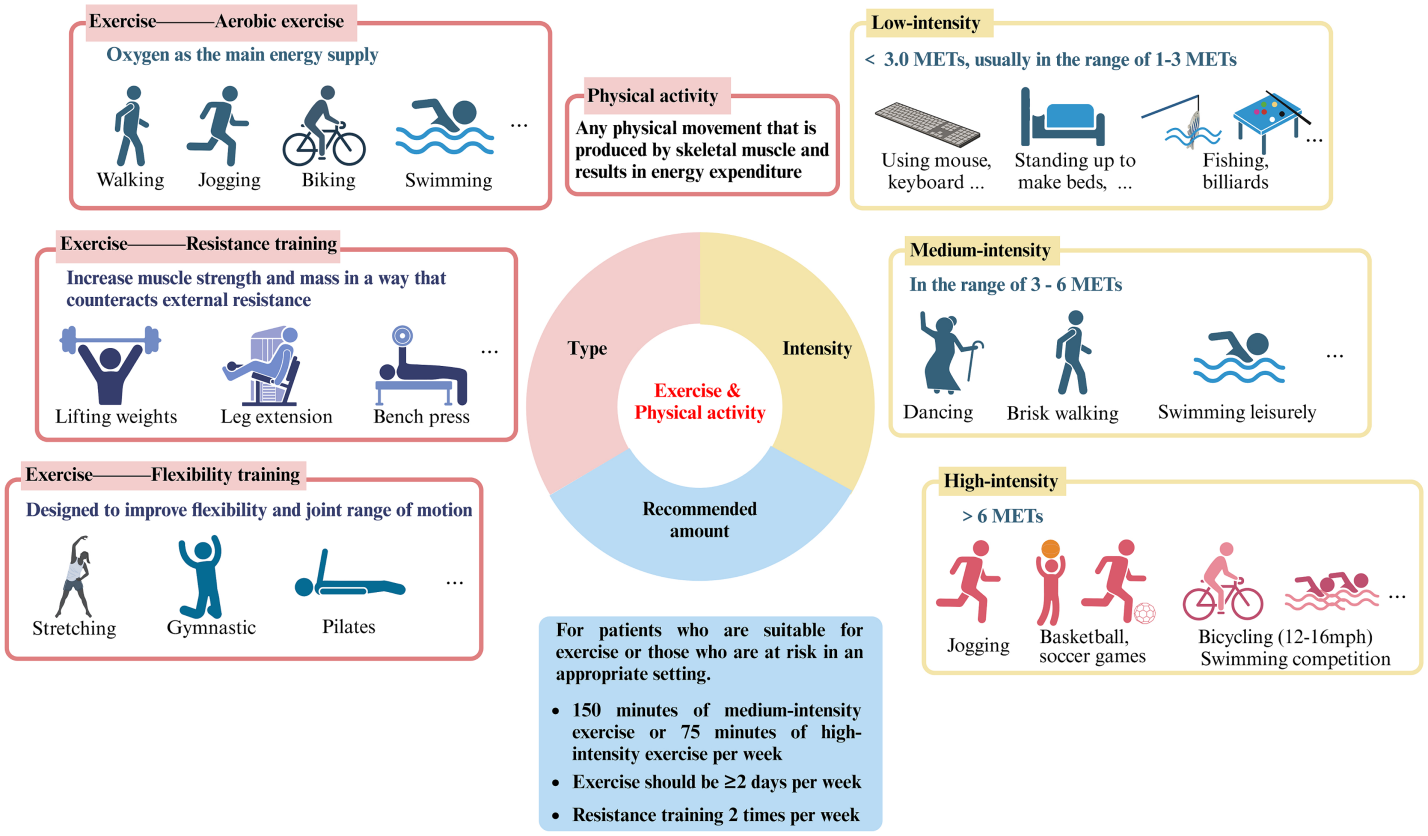
Type, intensity and recommended amount of exercise and physical activity (created with Biorender.com).

For sedentary people, the lack of specific knowledge about exercise is one of the main obstacles to the implementation of effective “exercise therapy,” including the differentiation and selection of the type and intensity of exercise (Figure 1).

### 2.1 Simple classification

The terms “exercise” and “physical activity” are frequently used interchangeably in literature. Nonetheless, a thorough analysis of these two concepts reveals that they are not precisely the same (12). Exercise refers to organized, planned physical activity that can be categorized into three groups: aerobic exercise, resistance training, and flexibility training. Aerobic exercise uses oxygen as the primary energy supply and includes activities such as running, cycling, swimming, brisk walking, and jumping rope. Resistance training, also known as strength training, involves countering external resistance to increase muscle strength and mass. This can include weight lifting, gymnastics, pull-ups, and the use of devices such as machines or rubber bands. Flexibility training is designed to increase flexibility and joint range of motion and includes activities such as yoga, Pilates, stretching, and gymnastics (13). Physical activity involves a variety of movements generated by skeletal muscles, leading to energy expenditure and encompassing an extensive array of occupational, recreational, and daily activities (12). However, overly detailed requirements and divisions have the potential to confuse and overwhelm individuals in regards to the implementation of “exercise therapy,” leading to a decrease in enthusiasm towards adhering to it. Based on the above explanations, it appears that various exercise modalities have been subsumed into the broader category of physical activity, potentially accounting for why the two terms are often used synonymously in existing literature. Therefore, this review does not make a specific distinction between the two.

### 2.2 Intensity

Variations in exercise intensity have been strongly associated with effects on ocular disease. The metabolic equivalent of task (MET) is a unit of measurement of the body’s energy expenditure at different levels of activity, with 1 MET representing the energy expenditure of a person at quiet sitting and rest, which is approximately 3.5 mcal/kg/min (14). It allows for the comparison of various forms of exercise and physical activity relative to energy expenditure during periods of rest, thus classifying the intensity of exercise, which is widely recognized and applied (15).

Low-intensity exercise has an energy expenditure of less than 3.0 METs, usually in the range of 1–3 METs, and specifically includes walking slowly (2.0), sitting and using hand tools such as mice and keyboards (1.5), standing for light physical activities such as making beds and washing dishes (1.5–2.5), playing pool (2.5), fishing (2.5), throwing darts (2.5), playing a musical instrument (2.0–2.5), etc.

Moderate-intensity exercise has METs between 3–6, comparable to a brisk walk which significantly accelerates the heart rate. Similar physical activities include washing windows, cars (3.0), sweeping floors (3.0–3.5), carrying & stacking wood (5.5), playing badminton recreationally (4.5), bicycling slowly on flat ground (6.0), dancing (3.0–4.5), playing table tennis (4.0), swimming leisurely (6.0), playing volleyball noncompetitively playing volleyball noncompetitively (3.0–4.0), etc.

High-intensity exercise, with jogging being a typical example, is defined as exercise greater than 6.0 METs, which can cause shortness of breath and a large increase in heart rate. Also included are carrying heavy loads such as bricks (7.5), heavy farming such as bailing hay (8.0), basketball game (8.0), bicycling on flat terrain with moderate (12–14 mph) or fast (14–16 mph) effort (8.0–10.0), cross country skiing (7.0–9.0), playing soccer (7.0–10.0), swimming with moderate to hard effort (8–11), playing competitive volleyball at the gym or beach (8.0), etc.

### 2.3 Modulation of ocular physiology by exercise and physical activity

The biological effects of exercise and physical activity extend across multiple facets and locations, positively impacting eye health by maintaining the health of normal physiological mechanisms of the eye and offering protection against diseases. Research indicates that exercise can increase tear production, contributing to the stability of the tear film and improvements in DED (16). In terms of corneal health, exercise regulates intraocular pressure, aiding in the maintenance of corneal health and mitigating acute corneal thinning and endothelial cell damage caused by acute high intraocular pressure (17). For the lens, there is a negative correlation between the intensity of physical activity and the degree of lens opacity (18); moderate exercise can reduce levels of inflammation and oxidative stress, slowing down the aging process of the lens (19). Exercise and physical activity also aid in weight control, reduces blood pressure and blood sugar levels, thus diminishing the impact of metabolic factors on the structure and function of the lens (20). The retina, with its intricate structure and complex functions, has a high blood flow and is susceptible to internal and external pathogenic factors. Exercise contributes to improving retinal microvascular blood supply and oxygen return, maintaining the health and functionality of retinal cells (21). Furthermore, exercise enhances the body’s antioxidative capacity and anti-inflammatory responses, protecting retinal cells from oxidative stress and inflammation, thereby reducing the risk of ocular diseases such as AMD and DR (22, 23). By boosting the expression of various neurotrophic factors, such as brain-derived neurotrophic factor (BDNF), exercise potentially offers retinal neuroprotection (24). Additionally, exercise can lower the risk of diabetes, hypertension, and other chronic diseases, preventing associated retinal pathologies such as DR.

### 2.4 Recommendations

According to the Global Recommendations on Physical Activity for Health and the United States Health and Human Services Physical Activity Guidelines for Adults, it is advised that adults engage in at least 150 min of moderate-intensity exercise or 75 min of vigorous-intensity exercise per week, with the possibility of combining both types based on individual circumstances (7, 25, 26). Exercise sessions should be spread across at least 2 days per week, and additionally, engaging in resistance training twice weekly is also recommended. Pertaining to eye health, the epidemiological evidence cited in this review primarily focuses on the positive impacts of moderate to vigorous-intensity exercise on specific ocular diseases, with the included study subjects being well-suited for physical activity. Consequently, we recommend that early to mid-stage patients of the ocular diseases listed in this review (who are in good physical condition) or high-risk individuals, persistently engage in long-term moderate to vigorous-intensity physical activities, such as running, walking, swimming, and cycling, in a suitable environment to potentially slow the progression of diseases or ameliorate symptoms. It should be noted that our recommendations come with certain prerequisites, as this review does not purport to suggest that exercise can serve as a direct treatment modality for specific ocular conditions. When afflicted with any eye disease, seeking medical assistance always takes precedence. Our aim is for patients to thoroughly assess their own conditions against the backdrop of medical treatment and consider maintaining physical activity and exercise as a supplementary means to assist treatment and improve their condition.

## 3 Effects of exercise and physical activity on ocular diseases

### 3.1 DED

#### 3.1.1 Effect of exercise and physical activity on DED

DED is a chronic disease affecting the ocular surface, caused by various factors and thought to be a functional unit disorder of the lacrimal gland, resulting in insufficient quantity and quality of tears to lubricate the eyes (27, 28). DED can cause persistent symptoms of irritation or burning in the eyes and vision impairment that negatively impacts daily life. Epidemiological studies have found that up to 75% of adults over the age of 40 may have DED, and it tends to worsen over time once it develops (29, 30).

In recent years, ample evidence has linked DED with lifestyle, highlighting the impact of exercise, diet, and sleep on its development (31). Two extensive cross-sectional studies based on questionnaires discovered that an increased level of exercise contributes to a reduced prevalence of DED. Individuals experiencing DED had reduced physical activity levels and increased sedentary behavior compared to those without DED (32, 33). Additionally, research indicates that physical activity can positively impact alleviating symptoms of DED. A cross-sectional study conducted among 944 web-based workers in China revealed that individuals who engaged in exercise at a minimum of three times per week experienced significantly reduced discomfort associated with their eyes, including decreased visual clarity (34). Another randomized controlled trial focusing on sedentary young adults in the office setting observed a significant decrease in discomfort symptoms, including eye pain, soreness, and eyestrain, after 3 months of exercise following an exercise guideline (35). These findings imply that exercise and physical activity may contribute to delaying the onset and slowing the progression of DED.

#### 3.1.2 Potential mechanisms

Numerous studies have demonstrated that loss of tear film homeostasis is the central mechanism of DED (31). In addition, tear hyperosmolarity, ocular surface inflammation, oxidative stress, ocular surface injury, and neurosensory abnormalities have all been identified as significant etiological factors in DED (36). Based on pathophysiological differences, DED can be classified into two categories: aqueous-deficient dry eye (ADDE) and evaporative dry eye (EDE) (29). Tear hyperosmolarity caused by decreased tear production (ADDE) or excessive evaporation (EDE) is one of the fundamental mechanisms of DED. Elevated osmolarity triggers apoptosis in epithelial cells and goblet cells, consequently reducing the lubrication ability of the ocular surface. This results in rapid tear film breakup and an additional rise in hyperosmolarity, thereby creating a self-perpetuating cycle. With increasing osmolarity, ocular surface damage increases and oxidative stress intensifies, eventually resulting in tear film homeostasis disruption (37). Studies have shown a temporary surge in tear volume and extended tear film rupture following aerobic exercise, indicating enhanced tear film stability (16). A prospective cross-sectional study involving 43 healthy participants found significant reductions in tear fluid levels of inflammation-related cytokine, including interferon-γ (IFN-γ), tumor necrosis factor-α (TNF-α), interleukin (IL)-1β, IL-4, IL-5, and dozens of others, after a 20 min exercise session in comparison to the baseline levels (38). More excitingly, exercise reduced the tear fluid concentration of the marker of oxidative stress, 8-hydroxy-2′-deoxyguanosine (8-OHdG), suggesting that exercise may play a role in alleviating DED by reducing the oxidative stress present in tears (39) (see Table 1).

**Table 1:** Effects of exercise and physical activity on common eye diseases: insights into myopia, cataract and DED with potential mechanisms explored.

Disease	Citation	Pre-clinical vs. clinical	Study design	Study population	Interventions	Study duration/follow-up period	Key findings	Potential mechanism
Myopia	Hansen et al. (40)	Clinical	Prospective cohort	1,443 participants	Physical activity (≥3 h/week)	16 years	Participating in three or more hours of physical activity per week significantly reduced the likelihood of developing myopia	(1) Increased release of dopamine facilitated by stronger light (outdoor exercise) (2) Reduction in time spent in near-work activities (3) Involvement of induced growth hormone in growth regulation of the eye
	Holton et al. (41)	Clinical	Cross-sectional	6,200 participants	Outdoor activity (≥1 h/day)	5 years	An hour of outdoor activity a day reduced the odds of being myopic by 13%	
	He et al. (42)	Clinical	Prospective cohort	1,903 schoolchildren	An additional 40 min class of outdoor activities	3 years	Cumulative incidence of myopia in intervention group is 9.1% lower than control group	
	French et al. (43)	Clinical	Cohort study	863 schoolchildren	Outdoor activity	6 years	The trend toward higher rates of myopia was more pronounced among children who spent less time outdoors	
	Lee et al. (44)	Clinical	Cross-sectional	5,048 male military conscripts	Outdoor activity	3 years	Less outdoor activity significantly associated with myopia prevalence	
	Ip et al. (45)	Clinical	Cross-sectional	2,339 schoolchildren	Outdoor activity	/	Outdoor exercise negatively correlates with myopia rates	
DED	Kawashima et al. (32)	Clinical	Cross-sectional	425 office workers	High, moderate or low PA	/	More Exercise, lower dry eye quality of life scores	(1) Improvement in tear film stability (by increasing tear volume and prolonging tear film breakup) (2) Reduction of tear inflammation and oxidative stress levels
	Kawashima et al. (35)	Clinical	Randomized controlled trial	36 office workers	Aerobic and resistance exercises	2 months	Higher level of physical activity was associated with a lower prevalence of DED	
	Cheng et al. (34)	Clinical	Cross-sectional	915 office workers	Physical activity	/	Higher level of physical activity was associated with fewer symptoms of DED	
	Nugyen et al. (46)	Clinical	Cross-sectional	48,418 subjects	Sedentary behavior	6 years	Higher sedentary time increased the risk of DED	
	Vera et al. (47)	Clinical	Quasi-experimental	19 helicopter pilots	Treadmill exercise	10 min	Exercise reduced tear osmolarity	
	Peart et al. (48)	Clinical	Quasi-experimental	12 subjects	Cycloergometer exercise	15 min	Exercise reduced ocular surface disease index	
	Sun et al. (39)	Clinical	Experimental	52 healthy and DED patients	Jogging	30 min	Exercise was associated with increased tear break-up time	
	Hanyuda et al. (33)	Clinical	Cross-sectional	102,582 participants	Physical activity	6 years	Lack of exercise linked to higher risk of DED among middle-aged and elderly in Japan	
Cataract	Williams (9)	Clinical	Prospective cohort	29,025 male and 11,967 female	Running	7 years	Vigorous physical activity delayed the onset of cataract	(1) Inhibition of oxidative stress and enhancement of antioxidant capacity (2) Suppression of inflammation and enhancement of anti-inflammatory capacity (3) Regulation of blood glucose, blood pressure and blood lipids
	Williams (11)	Clinical	Prospective cohort	32,610 runners and 14,917 walkers	Different levels of exercise	6.2 years	Moderate (walking) and vigorous (running) exercise were both significantly associated with lower cataract risk	
	Paunksnis et al. (18)	Clinical	Comparative	110 patients and 50 controls	Physical activity (>5,900 min/week)	/	The intensity of lens opacity and cataract are significantly related to physical activity	
	Zheng et al. (49)	Clinical	Prospective cohort	52,660 participants	Different levels of physical activity	12.1 years	High physical activity may be associated with decreased risk of age-related cataract	
	López-Sánchez et al. (50)	Clinical	Cross-sectional	17,777 participants	Physical activity	1 year	Physical activity was significantly associated with cataract	

### 3.2 Myopia

#### 3.2.1 Effect of exercise and physical activity on myopia

Myopia, or nearsightedness, is the prevalent refractive error that results in images being focused in front of the retina due to overstretching of the eye’s axis, thus making distant objects appear blurry (51). This condition is most prevalent during childhood and early adulthood (51). Myopia is a widespread ophthalmic disease globally, with an incidence of up to 80%–90% among young populations in certain regions of East and Southeast Asia (52). Its prevalence poses an escalating health issue worldwide. Any degree of myopia increases the likelihood of pathologic changes in ocular tissues, with a corresponding positive co-relation to the severity of myopia (53). High and pathological myopia substantially raise the likelihood of encountering vision-related complications such as glaucoma, retinal detachment, and macular degeneration (54). Therefore, preventing the onset of myopia and delaying its progression have been identified as crucial targets for public health interventions related to myopia (52).

Numerous studies have shown that physical activity positively affects the prevention of myopia and delays its progression (55–58). Those who engage in physical activity for over 3 h a week stand to reduce their chances of developing myopia by approximately 40% (40). One cohort study discovered a critical relevance between higher levels of physical activity and a reduction in the incidence of myopia, and that sedentary behavior increases the risk of myopia (59). Another longitudinal cohort study, based on a questionnaire, found that myopic students spend less time engaging in physical activity per day compared to nonmyopic students (60). Additionally, multiple studies have identified a substantial inverse relationship between time spent outdoors and the onset of myopia, with outdoor time being one of the primary protective factors against myopia (61, 62). A recent meta-analysis presented a notable nonlinear negative correlation between the time spent outside and the prevalence of myopia (63).

#### 3.2.2 Potential mechanisms

The specific mechanisms by which exercise and physical activity prevent and protect against myopia have not yet been clearly established. Some studies have proposed potential mechanisms. Physical activity and exercise can cause the choroidal layer to dilate by enhancing blood flow, potentially hindering eye axis lengthening (64). Studies on animals suggest that eye growth is suppressed when blood flow and choroidal thickness increase (65, 66). Exercise and physical activity may also impact myopia in non-visual ways by influencing vascular function, blood pressure, peripheral and central growth factors, insulin resistance, oxidative stress and inflammation (60). Prior studies have shown that exercise-induced growth hormone and these systemic mechanisms are involved in regulating ocular growth (60). Additionally, studies have shown that engaging in outdoor exercise is more effective in preventing and controlling myopia compared to indoor exercise (62). This is due to the stronger light environment outdoors, which is considered to be a crucial factor in myopia protection (62). Research on animals has demonstrated that myopic susceptibility and dopamine-related genes and proteins can be affected by light intensity and peripheral defocus (67). Among these, dopamine has been found to hinder ocular axis growth, and its protective effects can be counteracted by the presence of its antagonist, spiperone (68). Environmentally influenced peripheral defocus might also contribute to the emergence or progression of myopia (69, 70). When engaging in outdoor sports, objects are typically positioned farther away from the eyes, and refractive conditions are uniform, thus reducing peripheral defocus and decelerating eye axis growth to prevent myopia (53) (see Table 1).

### 3.3 Cataract

#### 3.3.1 Effect of exercise and physical activity on cataract

The lens is a transparent structure located at the front section of the eye that resembles a convex lens and possesses elastic properties. As one of the refractive media of the eye, the lens has the capacity to alter its shape due to its elasticity, enabling it to refract and focus light rays from varying distances onto the retina, consequently producing a sharp image (71). Cataract refers to the loss of clarity in the lens caused by clouding resulting from various factors (72). Globally, cataracts are a major contributor to visual impairment and blindness. Research indicates that this condition also increases the likelihood of depression and decreases life expectancy (73).

Long-term regular exercise and physical activity have demonstrated a favorable effect on the development and progression of cataract. Two prospective studies indicate that both moderate-intensity (walking) and high-intensity (running) exercise significantly reduce the risk of cataracts in men (9, 11) and women (9). Meanwhile, a case–control study identified an inverse relationship between lens cloudiness and the intensity of physical activity (18). Additionally, various detailed studies were conducted on different types, intensities, and durations of physical activity and exercise. The findings revealed a significant negative correlation between total physical activity and cataract incidence (49). Individuals who participated in rigorous physical labor experienced a 16% reduced risk of developing cataracts compared to those who led a sedentary lifestyle. Similarly, individuals who engaged in walking or biking for over 60 min daily recorded a 12% lowered prevalence of cataracts in comparison to those who rarely partook in such activities (49). Low-intensity physical activities, such as housework, did not seem to be associated with cataract risk (49). The evidence from these studies indicates that engaging in moderate to high intensity exercise and physical activity over a prolonged period can have a more clear-cut and recognized effect on reducing the risk of cataract.

#### 3.3.2 Potential mechanisms

##### 3.3.2.1 Enhancement of antioxidant defense in the lens

It is currently believed that reactive oxygen species (ROS) damage directly contributes to crystal clouding (72). Aging is an important driver of this process. In youth, antioxidants from the aqueous humor are sufficient to scavenge oxygen free radicals to prevent oxidative damage to the lens and, together with nutrients, maintain high metabolic activity of the lens, which is essential for normal clarity and refractive index (71). The levels of antioxidant enzymes, including dismutase (SOD) and glutathione peroxidase (GPX), in both the serum and lens notably decline with age (74). Furthermore, the level of reduced glutathione (GSH), which is the most critical antioxidant factor in the lens, decreases almost linearly and to an even greater extent (75, 76).

Research has indicated that consistent aerobic exercise aids in improving cellular capacity to fight against excessive build-up of ROS (77). Regular moderate-to high-intensity exercise increases the activity of endogenous antioxidant enzymes (SOD, GPX, and CAT), which improves the antioxidant capacity of the lens and slows down the aging process (78). However, this antioxidant effect does not appear to apply to all forms of exercise. For a single session of vigorous aerobic exercise, ROS and reactive nitrogen species are overproduced as a result of rapidly increased metabolism, which may instead induce oxidative stress and related injury (49). Therefore, finding the right form of exercise (regular and periodic exercise of moderate or higher intensity) is also crucial for improving lens turbidity.

##### 3.3.2.2 Suppression of inflammation

Inflammation is an additional crucial risk factor for cataract. Inflammation is intimately related to oxidative stress, and both tend to occur concurrently in multiple pathological conditions, such as cataract (79). An 11 years cohort study found that elevated plasma C-reactive protein (CRP) levels were significantly associated with the risk of developing cataract (80). Aging is a major factor in age-related cataract, and chronic inflammation is one of the hallmarks of aging (81). With aging, circulating levels of pro-inflammatory factors such as IL-6, TNF-α, and CRP generally increase (82). Regular exercise has been found to reduce circulating levels of IL-6 and CRP, which has been associated with exercise-induced upregulation of the anti-inflammatory factors lipocalin and IL-10 (83, 84). In addition, after 10–12 weeks of appropriate exercise in sedentary elderly subjects, circulating levels of TLR4 and other pro-inflammatory biomarkers were found to be reduced to levels similar to those in young adults (19). These findings suggest that chronic inflammation during aging may be ameliorated or even reversed by appropriate exercise, thereby decreasing the likelihood of cataract development or delaying the progression of crystalline opacity.

Analogous to oxidative stress, the modulation of inflammation by exercise and physical activity is strongly connected to the type and intensity of exercise and the physical state of the exerciser. Different studies on resistance and aerobic exercise have found that both have inhibitory effects on chronic inflammation, but aerobic exercise seems to be better suited to modulate the immune system and markers of inflammation in older adults than resistance exercise (85, 86). In contrast, prolonged resistance exercise did not produce positive anti-inflammatory effects in frail older adults.

##### 3.3.2.3 Amelioration of some systemic diseases

Various systemic diseases play a role in the onset and advancement of cataract. Men with hyperlipidemia (≥250 mg/dL) were discovered to have a heightened risk of developing cataract (87). Diabetic patients have a 5-fold increased risk of cataract compared to normal subjects (20). Case-control studies have identified a heightened risk of cataract in populations with hypertension, particularly in the case of posterior subcapsular cataract. Moreover, a linear relationship exists between blood pressure and the risk of cataract (88, 89).

The advantageous impacts of exercise and physical activity on regulating blood glucose, blood pressure, and blood lipid levels are well acknowledged. The American Diabetes Association (ADA) recommends adults with diabetes to participate in at least 150 min of moderate-intensity physical activity per week (90). A systematic review and meta-analysis suggests that a combination of moderate-to high-intensity aerobic exercise and resistance training followed by regular exercise (≥4 weeks) reduces blood pressure by approximately 2–5 mmHg in normotensive adults and 5–7 mmHg in hypertensive adults (91). Exercise and physical activity are now thought to improve hyperlipidemia by modulating plasma lipoprotein levels, and a randomized controlled clinical trial found a significant increase in plasma high density lipoprotein (HDL) (4.4 mg/dL) in the exercise group after 1 year of regular exercise (running at least 8 km per week) (92).

Similarly, the extent to which exercise improves systemic disease is strongly linked to the duration and intensity of exercise. In hyperlipidemia, for example, a significant correlation was found between weekly exercise distance and low-density lipoprotein cholesterol (*r* = −0.31), plasma HDL cholesterol (*r* = 0.48) and HDL2 (*r* = 0.41). In addition, regular exercise for at least up to 9 months was required to observe significant changes in lipid profiles (93, 94) (see Table 1).

### 3.4 Glaucoma

#### 3.4.1 Effect of exercise and physical activity on glaucoma

Glaucoma is the primary cause of irreversible vision loss globally (95). As a classification of neurodegenerative diseases, is characterized by the gradual degeneration of retinal ganglion cells (RGCs) and axons (96). Degeneration of the optic nerve results in cupping, which is the distinctive appearance of the optic disc and accompanying loss of vision (97). Intraocular pressure (IOP) is the most significant, and the only controllable risk factor for glaucoma, so reducing IOP through medication or surgery is currently the mainstay of glaucoma treatment (98). However, some clinical studies have found that glaucoma patients continue to progress even when IOP is lowered (99).

In recent years, numerous studies have disclosed that modifiable environmental factors, such as exercise, smoking, alcohol consumption, and nutrition, possess a significant impact on the development of glaucoma (100). Long-term, regular exercise and physical activity have been demonstrated to reduce the incidence of glaucoma or slow its progression (8, 101, 102). A prospective study including 9,519 adults for up to 5 years discovered a significantly lower occurrence of glaucoma in those who abided by physical activity guidelines compared to those who did not engage in exercise (2.24% versus 1.14%) (8). A further retrospective analysis examined 24 patients with primary open-angle glaucoma (POAG) or pseudoexfoliation glaucoma and found that those who engaged in exercise habits (≥30 min per week) experienced less glaucomatous visual field deterioration compared to these non-exercising counterparts (10).

A considerable amount of research suggests that exercise and physical activity have the potential to prevent and mitigate various forms of damage to RGCs and optic nerves (103). A cross-sectional study with over 40,000 participants discovered that increased levels of physical activity led to an augmentation of the thickness in the plexiform layer within the retina (104). Experimental studies offer more unambiguous evidence. An experiment investigating age-related optic nerve injury revealed that regular aerobic exercise (swimming for 60 min per day, 5 days per week for 6 weeks) led to similar responses to acute ischemia-reperfusion-induced optic nerve injury in older mice (12 months) as compared to non-exercised young mice (3 months) (105). Additionally, exercise reduced injury-induced retinal neuroinflammation in aged mice. Another study on injury caused by optic nerve severance demonstrated that retinal ganglion cell survival significantly increased following a 4 weeks period of aerobic exercise, specifically running at 9 m/min for 30 min per day (106).

The effect of exercise and physical activity on glaucoma is intricately linked to the type and intensity of exercise undertaking. In a substantial 7.7 years cohort study comprising over 29,854 male runners belonging to running clubs located throughout the United States, an evident dose-dependent correlation was discovered between exercise patterns and glaucoma hazards, with swifter running speeds and longer running distances demonstrating a decreased risk of glaucoma (107). Another retrospective study involving 10,243 South Korean men aged 40 or older uncovered an inverse relationship between the development of glaucoma and engagement in medium-to-high intensity physical activity (108). Furthermore, a longitudinal study observing 141 patients with glaucoma discovered that the rate of visual field loss decreased with increased moderate to high activity levels (according to accelerometer measurements) (109). However, another study revealed that low and high-intensity exercise might elevate the risk of glaucoma in comparison to the moderate-intensity exercise recommended by the American College of Sports Medicine (ACSM) guidelines (110). The study also uncovered that undertaking high-intensity exercise daily was linked to a greater occurrence of glaucoma than exercising three times weekly. It is noteworthy that not all types of exercise and physical activity are advantageous for individuals with glaucoma. The study has demonstrated that engaging in vigorous isometric workouts such as wall push, suspension training, resistance band training, among others, as well as performing exercises in inverted body positions, and playing high-resistance wind instruments such as trumpet, trombone, French horn, and others have been shown to elevate IOP, thereby elevating the risk of developing glaucoma (111, 112).

It should be noted that there are multiple types of glaucoma, and exercise and physical activity may be considered a risk factor for certain types, such as pigmentary glaucoma. Pigmentation of corneal endothelial cells occurs in patients with pigment dispersion syndrome. When pigment particles enter the trabecular meshwork endothelial cells, they can gradually lead to pigment overload and death of the trabecular meshwork cells. This, in turn, causes trabecular collapse, obstruction of atrial aqueous outflow pathways, and increased intraocular pressure. Ultimately, this can lead to pigmentary glaucoma (113). Studies have shown that after aerobic exercise, patients with pigmented dispersion syndrome and pigmentary glaucoma experience a dispersion of pigments in the anterior chamber that is significantly increased (114, 115). A patient with pigment dispersion syndrome presented with typical symptoms of elevated IOP, blurred vision, and halos after strenuous exercise (116). However, after expanding the sample size, it was found that exercise did not significantly increase clinical IOP in patients with pigmentary glaucoma (117). Iris depressions have been suggested as a possible mechanism for pigment release (118). Patients with pigmentary glaucoma experience a significant increase in iris depressions following exercise (119). Research has demonstrated that exercise can increase the IOP pulse and affect atrial fluid dynamics, resulting in enhanced cyclic atrial fluid flow through the pupil. This, combined with reverse pupillary obstruction, can cause intermittent excessive iris depression, leading to an increase in pigment dispersion (119). Although current research does not suggest that all patients with pigmentary dispersion syndrome or pigmentary glaucoma should avoid all forms of exercise, highly stimulating exercise should be avoided as much as possible. It is necessary for these patients to carefully consider their condition and seek evaluation by a healthcare professional prior to exercising at a low or moderate intensity (114).

#### 3.4.2 Potential mechanisms

##### 3.4.2.1 Regulation of IOP

Excessive IOP can compress, deform, and alter the sieve plate on the inner eye wall, leading to mechanical axonal damage and transit disorders that impede the brainstem’s provision of trophic factors to RGCs (120). When IOP exceeds ocular perfusion pressure (OPP), blood supply to the optic nerve is restricted (121). The various factors listed above ultimately lead to the demise of RGCs and initiate the onset of glaucoma. Lowering IOP is a crucial factor in preventing and delaying the progression of glaucoma through exercise and physical activity (122). According to a randomized controlled study, a notable decrease in IOP was observed among healthy adults who engaged in 20 min of aerobic exercise with a return to baseline levels in approximately 1 h (123). Furthermore, post-exercise IOP levels decreased by 2.7 mmHg in a study of resistance training (124). Another prospective, interventional study found that aerobic exercise reduced the baseline level of IOP in subjects with glaucoma by 4.6 ± 0.4 mmHg after 3 months, and this reduction was maintained for 3 weeks (125). Therefore, examining the specific mechanisms by which exercise improves glaucoma requires an examination of how exercise regulates IOP. Maintaining normal intraocular pressure depends on a balanced interplay between the production of aqueous humor by the ciliary body and its drainage through the trabecular meshwork and uveoscleral outflow pathway (96). The present study suggests that acute exercise may alter IOP by, among other things, decreasing blood pH, increasing plasma osmolality, increasing blood lactate, decreasing norepinephrine concentration, increasing the nitric oxide/endothelin ratio (resulting in increased NO production and decreased endothelin content), stimulating β2 receptors, and altering ocular blood flow after exercise (122). Different types of exercise have been found to have varying effects on IOP regulation. Isometric exercise has primarily been associated with hyperventilation and hypocapnia, resulting in a decrease in IOP, while isotonic exercise has been primarily linked to an increase in colloid osmolality (126–128). However, research on the mechanisms by which long-term exercise and physical activity impact baseline levels of IOP is lacking, which suggests an avenue for future research.

##### 3.4.2.2 Inhibition of oxidative stress and inflammation in the trabecular meshwork and retina

Oxidative stress, cellular senescence, and mitochondrial dysfunction increase in the aging retina and are significant risk factors for glaucoma (131). Oxidative stress and lipid peroxidation are principle initial contributors to the onset of para-inflammation in the retina (132). ROS activate retina-resident macrophages and neuroimmune cells, such as microglia and astrocytes, which release proinflammatory mediators and trigger neuroinflammation, ultimately accelerating the death of RGCs (132). Moreover, the trabecular meshwork serves as the “gateway” of aqueous humor outflow in the eye and is a highly vulnerable tissue to oxidative stress. This stress disrupts the normal drainage of aqueous fluids and ultimately leads to increased intraocular pressure and damage to the optic nerve (131). Research has shown that individuals with glaucoma exhibit elevated levels of oxidative stress markers, including 8-OH-dG and malondialdehyde, within their aqueous humor, trabecular meshwork, and serum (133, 134).

Prolonged aerobic exercise has been demonstrated to reduce oxidative stress and enhance antioxidant capacity in elderly individuals (135). An experimental study demonstrated a reduction in levels of oxidative stress within the retinas of mice after 12 weeks of regular bench running exercise (5–12 m/min, 30–60 min/day, 3 days/week) (130). This provides more direct evidence of the benefits of aerobic exercise. Previous studies have revealed that aerobic exercise reduces inflammatory factors in the bloodstream, spinal cord, cortex, and hippocampus, such as IL-1, IL-6, and TNF-α (136). At present, there is no direct evidence indicating that exercise ameliorates retinal inflammation, it has been found that exercise-induced myokine Irisin can inhibit oxygen-induced pathological angiogenesis, inflammation, and apoptosis in the retina *in vivo* (137).

##### 3.4.2.3 Improvement of mitochondrial function

Mitochondria contribute to oxidative stress in living organisms. Mitochondrial dysfunction is a critical aspect of aging, characterized by changes in biogenesis, protein inhibition, mitochondrial autophagy, and kinetics (138, 139). The retina is among the most metabolically active tissues within the body and contains a substantial quantity of mitochondria. These mitochondria are primarily located in the myelin-free axons of RGCs and the inner segments of photoreceptors, fulfilling a crucial role in normal energy transportation (140). Mitochondrial dysfunction, potentially caused by various mechanisms such as dysregulation of signaling, dysfunction of pathways, genetic variations, and oxidative stress, is significantly associated with a majority of blinding eye conditions, including glaucoma (141, 142). The mitochondria in the RGCs are the first to be affected by glaucoma (143). Furthermore, the expression of OPA 1, a gene that is present in RGCs soma and axons and linked to both spontaneous and hereditary mitochondrial optic neuropathy, exhibits noteworthy down-regulation in patients identified with POAG, providing evidence of a genetic association connecting mitochondrial function and POAG (144). Mitochondrial abnormalities in morphology, reduced mass, decreased oxidative capacity, and abnormal autophagy, among other factors, can all contribute to glaucoma development (142).

Numerous studies indicate that aerobic exercise boosts the mitochondrial biosynthetic pathway and cellular respiratory capacity, leading to improved mitochondrial mass, structure, and function. Exercise improves an organism’s resilience against inhibitors of mitochondrial complex I, which is the initial enzyme-protein complex in the mitochondrial OXPHOS pathway. The downregulation of this complex may result in mitochondrial dysfunction. Physical activity also boosts the expression of mRNAs associated with mitochondrial complex I (145). Experimental studies have demonstrated that aerobic exercise can preserve mitochondrial metabolism by preventing age-related mitochondrial damage in skeletal muscle, achieved through inhibiting mitochondrial schizopyranin expression in a PGC-1α-dependent manner (146). Other studies have indicated that aerobic exercise enhances mitochondrial biogenesis and function by activating Nrf2 (139). Elevated mRNA levels of lysosome-associated membrane protein 2 (LAMP-2), Atg and LC3II in lateral femoral muscles was found in overweight older women after 6 months of weight loss and moderate-intensity exercise. Additionally, this study discovered that lifelong physical activity preserves LC3II and Atg at youthful levels (147). This indicates that physical activity enhances mitochondrial autophagy and consequently attenuates mitochondrial dysfunction. However, further validation is necessary to determine whether these particular mechanisms apply to the optic nerve injury model of glaucoma.

##### 3.4.2.4 Facilitating the release of protective factors

###### 3.4.2.4.1 BDNF

BDNF, a protein from the neurotrophic factor family, is produced by retinal neurons like RGCs, anaplastic synaptic cells, retinal neuroglia (Müller cells), astrocytes, and photoreceptors (148). It has been shown that BDNF protects RGCs’ survival in pathological conditions such as glaucoma and optic nerve injury (149). BDNF can enhance the retina’s resistance to injury by regulating the formation and maintenance of retinal neural circuits. Studies have shown that exercise can effectively prevent complement-mediated synapse loss (150). This helps to resist neurodegenerative changes in the structure, function, and molecules of the inner retina caused by glaucoma (151, 152). Currently, BDNF is widely recognized as a pivotal factor that mediates the impact of exercise on varying forms of retinal damage (153). Aerobic exercise, including long-term endurance training and short-term acute exercise, enhances circulating levels of BDNF in both healthy and chronically ill individuals. However, resistance training did not have such an effect (154). Experimental studies have found that damaged retinas of untrained mice had significantly decreased levels of BDNF, whereas retinal BDNF levels were significantly increased in trained mice (24). The primary mechanism of BDNF action relates to its binding to the TrkB receptor, which triggers the initiation of numerous signaling cascades. Activated TrkB enhances Erk1/2, which are responsible for retinal ganglion cell (RGC) survival, while concurrently inhibiting glycogen synthase kinase 3β (GSK3β) activity through enhancing PI3K/Akt signaling, thereby promoting neuronal axon growth and cell survival (152, 155). Inhibiting TrkB effectively impairs the ameliorative effects of exercise on retinal damage in disease models, including glaucoma and DR (150). In a mouse model of glaucoma, administration of BDNF resulted in increased survival of the RGC and improved retinal function (156).

Furthermore, insulin-like growth factor-1 (IGF-1) and neurotrophic growth factor (NGF) have also been validated for their neuroprotective effects in cases of retinal injury (103). Nonetheless, it is still unclear if they contribute to the protective effects of exercise, necessitating further comprehensive investigation.

###### 3.4.2.4.2 Dopamine

Dopaminergic neurons are considered a crucial component of the motor system, with physical exercise and activity known to stimulate dopamine release (157, 158). Extensive animal studies have demonstrated that regular aerobic activities such as running and swimming lead to significant increases in serum and brain dopamine levels in mice or rats (158, 159). The dopaminergic system’s role in regulating the balance of aqueous humor dynamics, a key pathophysiological process in glaucoma, has been widely studied since the 1980s. Dopamine’s activity is mediated primarily through five G protein-coupled receptors belonging to two subfamilies: the D1-like receptors (D1R and D5R) and the D2-like receptors (D2R, D3R, and D4R) (160). Activation of D2R and D3R helps lower intraocular pressure (161–164). Many classic D2R agonists, including bromocriptine, cabergoline, lisuride, and lergotrile, have been used to reduce intraocular pressure in humans and animals (160). In 2000, a study on the selective D3R agonist 7-OH-DPAT revealed its potential role in reducing rabbit intraocular pressure, marking the first indication of D3R’s involvement in lowering eye pressure (163). Subsequent research on various D3R agonists and D3R^−/−^ mice further confirmed this view (165, 166). The presence of D3R in the sympathetic nerve fibers of the ciliary body suggests that D3R activation could block the inflow of aqueous humor, thereby reducing intraocular pressure (162). A recent study by Reyes-Resina et al. (167) found that D3R expression in the ciliary body slices of glaucoma patients was significantly lower than that in controls, although the sample size of two was too small for statistical analysis, the findings undeniably further elucidate the connection between the dopaminergic system and glaucoma. While D2R and D3R have been detected in the human retina, there is a lack of research on abnormalities in the dopaminergic system within the retina of glaucoma patients. However, a study on a quail model of glaucoma found a reduction in dopaminergic cells and dopamine rhythms in the retina following glaucoma (168). In summary, physical exercise and activity may positively impact glaucoma by promoting dopamine release and its interaction with D2R and D3R. Nonetheless, this hypothesis requires further substantiation through more in-depth and direct evidence (see Table 2).

**Table 2:** Effects of exercise and physical activity on common eye diseases: insights into glaucoma with potential mechanisms explored.

Disease	Citation	Pre-clinical vs. clinical	Study design	Study population	Interventions	Study duration/follow-up period	Key findings	Potential mechanism
Glaucoma	Yan et al. (123)	Clinical	Paired comparative	29 healthy participants	Jogging	20 min	A decrease in IOP after aerobic exercise in healthy subjects	(1) Decrease in intraocular pressure (decreases blood pH, increases plasma osmolality and blood lactate, decreases norepinephrine concentration…) (2) Inhibition of oxidative stress and inflammation
	Yeak et al. (129)	Clinical	Prospective, interventional cohort	45 healthy participants	Aerobic exercise and strength training	6 weeks	Regular exercise significantly reduces intraocular pressure in healthy people	
	Meier et al. (8)	Clinical	Prospective cohort	9,519 participants	Physical activity	5.7 years	Higher level of physical activity was associated with a lower risk of incident glaucoma	
	Yokota et al. (10)	Clinical	Retrospective cohort	24 POAG patients	Exercise (≥30 min/week)	3 years	Glaucoma progresses slower in patients with self-reported exercise habits	
	Madjedi et al. (104)	Clinical	Cross-sectional	3,627 participants	Physical activity	5 years	Higher overall PA level was associated with thicker mGCIPL	
	Williams et al. (107)	Clinical	Prospective epidemiologic cohort	29,854 male runners	Running	7.7 years	Vigorous physical activity may reduce glaucoma risk	
	Seo et al. (108)	Clinical	Cross-sectional	10,243 men	Different intensity of exercise	5 years	Exercise intensity negatively correlates with odds of developing glaucoma	
	Lee et al. (109)	Clinical	Longitudinal, observational study	141 participants	Different intensity of exercise	1 week	Moderate-to-vigorous physical activity was associated with slower visual field loss in glaucoma patients	
	Lawson et al. (24)	Pre-clinical	Randomised controlled	48 mice	Treadmill exercise (5 days/week, 60 min, 10 m/min)	2 weeks	Exercise increased retinal BDNF protein levels by 20% compared with inactive mice	(3) Improvement of mitochondrial dysfunction (4) Increased protective myokine expression (BDNF, Irisin…)
	Kim et al. (130)	Pre-clinical	Randomised controlled	24 mice	Treadmill exercise (3 days/week, 30–60 min, 5–12 m/min)	12 weeks	Regular exercise can reduce retinal oxidative stress	
	He et al. (106)	Pre-clinical	Randomised controlled	24 mice	Treadmill exercise (5 days/week, 30 min, 9 m/min)	4 weeks	Treadmill training effectively rescues RGCs that develop neurodegeneration after optic nerve transection	
	Chrysostomou et al. (105)	Pre-clinical	Randomised controlled	30 mice	Swimming (5 days/week, 60 min)	6 weeks	Exercise protects the aged optic nerve against functional loss	

### 3.5 DR

#### 3.5.1 Effect of exercise and physical activity on DR

DR is the leading retinal vascular ailment that results in visual impairments like floaters, distorted vision, blurry vision, and in more severe cases, retinal detachment that can cause total or partial vision loss (169). DR can also affect the macula and lead to diabetic macular edema (DME). DME is featured by vascular leakage and results in swelling of the macula, which is the primary reason for blindness among diabetic patients (170).

Clinical studies have confirmed that regular exercise or physical activity and maintaining a healthy diet can significantly reduce the risk of developing diabetes and related ocular complications (171). A retrospective study of 3,031 adults demonstrated that individuals who consistently engaged in higher levels of physical activity over an extended period had a lower incidence of DR (172). Conversely, a sedentary lifestyle was found to significantly increase the likelihood of diabetic patients developing DR (173). Another pre-and post-clinical trial conducted on patients with DR indicated significant reductions in fasting blood glucose and central macular thickness in participants who underwent long-term moderate-intensity aerobic exercise regularly (45 min, three times per week for 12 weeks) (174). Basic research has provided further evidence that 8 weeks of treadmill exercise (8 m/min, 30 min per day, 6 days per week) yielded a significant reduction in retinal vascular endothelial growth factor (VEGF) expression and retinal cell apoptosis in diabetic mice models (175).

The intensity of exercise and physical activity is associated with their ameliorative effect on DR. Previous research suggests that the benefits of moderate-intensity aerobic exercise and physical activity for DR are greater than those of low-and high-intensity exercise (176). High-intensity interval training (HIIT) has been found to enhance choriocapillaris perfusion in healthy individuals. Nevertheless, clinical trial studies have not observed any ameliorative effects of HIIT on the microvasculature in patients with type 1 diabetes (T1D) (177, 178). At present, research on the influence of exercise and physical activity on DR primarily concentrates on the advantages of aerobic exercise. Additional studies are necessary to explore and showcase the impacts of other forms of exercise and physical activity on DR.

#### 3.5.2 Potential mechanisms

##### 3.5.2.1 Regulation of blood glucose

Briefly, DR is a microangiopathy with a complex pathophysiology and unclear pathogenesis (179). Various studies have established a correlation between hyperglycemia and retinal microvascular damage, indicating the former as a major and direct cause (180). Hyperglycemia leads to several pathophysiological alterations, such as oxidative stress, accumulation of sorbitol and advanced glycosylation end-products (AGEs), activation of protein kinase C (PKC), chronic neuroinflammation, and others (181). These pathways result in dysfunction of the vascular endothelium of the retina, marked by the loss of pericytes and endothelial cells, thinning of the basement membrane, increased retinal permeability, and the absence of neuroprotective molecules (182, 183). Retinal vascular endothelial dysfunction worsens retinal hypoxia, neuronal dysfunction, and leads to the development of various angiogenic factors such as VEGF, growth hormone-insulin-like growth factor (GH-IGF), and erythropoietin. Ultimately, this results in retinal neoangiogenesis, which is a typical feature of DR and a hallmark of its progressive development (182, 184).

The effectiveness of exercise and physical activity in improving blood glucose levels has been well-established through numerous studies. Different types of physical activity, like aerobic exercise, resistance training, and high-intensity interval exercise (HIIE), have been found to boost blood glucose levels in patients with type 2 diabetes by augmenting insulin sensitivity, promoting mitochondrial function, and adjusting metabolic parameters such as blood pressure and blood lipids (185–191). It has been found that combined exercise produces a more significant effect on blood glucose intervention than any single type of exercise alone (192, 193). A meta-analysis on resistance training demonstrated that high-intensity resistance training is more effective than low or moderate-intensity for reducing insulin levels and overall glycemic management (189). Furthermore, numerous studies indicate that engaging in exercise after a meal is more effective in regulating glycemic levels by reducing sudden spikes in blood glucose. The advantages are particularly evident for exercise sessions lasting 45 min or longer (188, 194).

##### 3.5.2.2 Retinal neuroprotection

A rising number of studies have shown that pure microvascular injury cannot fully elucidate the pathogenesis of DR. Neuroretinal damage is found to be equally indispensable in DR’s development, even before microvascular injury (195). Diabetes mellitus influences the functioning and structure of all retinal cell types, and an increased expression of apoptotic markers of RGCs (caspase-3, Fas, and Bax) was noticed in diabetic patients’ retinas (196). Animal studies show that hyperglycemia speeds up retinal neuron apoptosis in mice, while also promoting glial cell activation and impaired metabolism (195). Additionally, a rat model of diabetes-induced early retinal neuropathy showed reduced BDNF levels, which further increased aberrant autophagy (197). BDNF upregulation has been suggested as a possible therapeutic avenue for DR, as it protects retinal cells by restoring proper autophagic responses in the retina (198).

As previously described, exercise and physical activity enhance the expression of multiple neurotrophic factors, including mainly BDNF, exhibiting greater levels, particularly in the retina and serum (24). BDNF has the potential to mitigate DR by promoting neuroretinal repair and survival caused by ischemia through the activation of TrkB and corresponding downstream pathways (199, 200).

##### 3.5.2.3 Inhibition of hyperglycemia-induced oxidative stress and inflammation in retinal vessels

Oxidative stress is a significant contributor to the development of DR due to hyperglycemia leading to ROS overproduction (201). Oxidative stress causes damage to the cytoarchitecture and mitochondria, leading to apoptosis and depletion of pericytes (one of the major cell types of the retinal microvasculature, which controls endothelial cell proliferation and protects endothelial cells from lipid peroxide-induced damage), ultimately triggering or exacerbating the onset and progression of DR (202–204). Additionally, oxidative stress activates the inflammatory cascade. It has been found that hyperglycemia can lead to abnormal signaling pathways, among them phosphoinositide 3-kinase/Akt/protein kinase B and inducible NOS (iNOS). One specific manifestation is the inhibition of eNO synthase (eNOS) activation and the promotion of up-regulation of inflammatory mediators such as iNOS, IL-6, and TNF-α (205). The paracrine inflammatory response is activated, causing abnormal interactions between leukocytes and endothelial cells, ultimately resulting in retinal microvascular damage (132).

Exercise has been found to provide protection to retinal cells from damage caused by diabetes by inhibiting inflammatory and oxidative stress that are fundamental to the disease (22). For DR, Cheng et al. (175) reported that engaging in long-term regular platform running exercise could suppress vascular inflammation and oxidative stress in diabetic mice by upregulating miR-181b through activation of AMPK, further improving endothelial dysfunction in DR. Another study, based on a survey of 157 patients with retinopathy, found a negative correlation between moderate to high intensity physical activity and systemic inflammation (206).

##### 3.5.2.4 Others

Chronic hyperglycemia-induced retinal microvascular pathological changes and structural abnormalities can result in hemodynamic abnormalities that could facilitate DR progression (183, 207). Studies have shown that exercise may improve retinal hemodynamics by promoting the expression of related adipokines, such as lipocalin, in the plasma and retina. Fundamental research has discovered that lipocalin impedes tube formation in human retinal microvascular endothelial cells. This suggests that lipocalin could be a potential treatment target for angiogenesis in DR (208). In a clinical study involving patients diagnosed with type 2 diabetes, researchers found that an increase in plasma lipocalin levels was positively associated with retinal vessel diameter, retinal blood velocity and flow, while being negatively associated with the total peripheral resistance of retinal arteries (209).

Furthermore, exercise and physical activity may improve DR by influencing 25-hydroxyvitamin D (25 OH-D) levels. A wealth of evidence supports the notion that enhanced physical activity leads to better 25 OH-D status in individuals of all ages, which is linked to microvascular events (210–212). It has been shown that the impact of physical activity on fasting blood sugar levels may be influenced by the genotype of the vitamin D receptor (213) (see Table 3).

**Table 3:** Effects of exercise and physical activity on common eye diseases: insights into DR and AMD with potential mechanisms explored.

Disease	Citation	Pre-clinical vs. clinical	Study design	Study population	Interventions	Study duration/follow-up period	Key findings	Potential mechanism
DR	Wang et al. (214)	Clinical	Retrospective cohort	3,031 adults	Different levels of physical activity	/	More physical activity and less sedentary lifestyles associated with lower prevalence of DR	(1) Regulation of blood glucose, blood pressure and blood lipids (2) Inhibition of oxidative stress and inflammation (3) Improvement in hemodynamics (4) Regulation of BDNF, 25 (OH)D
	Loprinzi et al. (173)	Clinical	Cross-sectional	282 diabetic patients	Sedentary behavior	1 year	Sedentary lifestyle significantly increased the likelihood of diabetic patients developing DR	
	Soleimani et al. (174)	Clinical	Before-after clinical trial	40 DR subjects	Moderate-intensity aerobic exercise (3 times/week, 45 min)	12 weeks	Lower FBS (mg/dL) and CMT (microns) in moderate-intensity aerobic exercise group	
	Cheng et al. (175)	Pre-clinical	Randomised controlled	30 mice	Running exercise	8 weeks	Chronic exercise alleviates endothelial dysfunction, vascular inflammation, and oxidative stress	
AMD	Knudtson et al (215)	Clinical	Prospective cohort	3,874 participants	Physical activity	15 years	Physical activity reduced the risk of exudative AMD by 70% in 15 years	(1) Inhibition of oxidative stress and inflammation
	Ulańczyk et al. (216)	Clinical	Cross-sectional	330 AMD cases and 121 controls	Physical activity	/	Physical exercise might delay AMD progression and help retain better visual function	
	McGuinness et al. (23)	Clinical	Prospective cohort	41,514 participants	Vigorous exercise (frequent ≥3 times/week and less frequent 1–2 times/week)	6 months	Vigorous exercise is protective against moderate AMD in women	(2) Production of adipokines promotes
	Zhang et al. (217)	Pre-clinical	Randomised controlled	20 mice	Exercise	5 weeks	Exercise partially preserves retinal function	
	Cui et al. (218)	Pre-clinical	Randomised controlled	22 mice	Treadmill running (6 times/week, 60 min, 15 m/min)	4 weeks	Treadmill training protects laser-induced CNV and enhances anti-angiogenic efficacy	

### 3.6 AMD

#### 3.6.1 Effect of exercise and physical activity on AMD

The macula is situated in the posterior central portion of the retina, representing the most visually acute part of the retina. AMD is a degenerative condition of the macula caused by the malfunction of retinal pigment epithelium (RPE) and the death of photoreceptor cells (219). AMD is the primary cause of irreversible vision impairment in individuals aged 60 and above. Advanced AMD can drastically diminish visual acuity, impacting overall quality of life (220). Clinically, AMD has two subtypes: non-neovascular and neovascular (219). Between 80 to 85% of patients are diagnosed with non-neovascular AMD, characterized by the loss of photoreceptors and RPE cells, and exposure of the underlying choroidal vessels, generally leading to a better visual prognosis. Conversely, neovascular AMD, marked by the formation of new blood vessels beneath the RPE, within the retina, or under the retina, accounts for less patient numbers but is responsible for nearly 80% of severe vision loss associated with AMD (221, 222).

Age is considered the primary factor in AMD, while smoking is the top modifiable risk factor associated with AMD (219). However, several studies have also confirmed the correlation between exercise and AMD. A study spanning 15 years and including 3,874 adults indicated a marked reduction in the incidence of neovascular AMD among those who maintained an active lifestyle (exercising at least thrice weekly) over an extended period, compared to those who remained inactive. After adjusting for variables, the study discovered that incorporating more daily walks into one’s routine can reduce the risk of developing AMD (215). A sedentary lifestyle can exacerbate AMD progression, whereas patients who engage in regular exercise and physical activity tend to have better vision (216). Additionally, a systematic review and meta-analysis determined that physical activity is linked to a decreased incidence of both early and late-stage AMD (223). Animal studies indicate that aerobic exercise may safeguard photoreceptor cells and RPE from harm and hinder reduction in retinal and photoreceptor layer thickness in a mouse model of retinal degeneration (217).

#### 3.6.2 Potential mechanisms

Various risk factors for AMD, such as age, smoking, diet, sun exposure, and alcohol consumption, can induce or exacerbate a common pathophysiologic process known as oxidative stress at the molecular level (224). The macula ensures high metabolic activity to safeguard the large amount of energy required for light sensing and visual signal processing by the cone cells. This finding suggests that the macula is exposed to high levels of ROS, making it more susceptible to damage from oxidative stress (224). In atrophic AMD, accumulated ROS directly damage the cell membranes, DNA, and proteins of RPE cells and photoreceptor cells, leading to structural and functional impairments. Oxidative damage also results in the accumulation of cellular metabolic byproducts like lipofuscin within the RPE cells, further exacerbating cellular dysfunction and death by disrupting autophagy and affecting normal metabolism (222, 225). VEGF-mediated abnormal vascular generation is a key factor inducing CNV in late-stage neovascular AMD. The NF-κB pathway, activated under oxidative stress, translocates to the nucleus, directly enhancing VEGF gene transcription and stimulating neovascularization (226, 227). Signaling pathways such as MAPK and PI3K/Akt are also involved in the regulation of VEGF by oxidative stress (228, 229). ROS clearance is a popular research topic in AMD treatment. Studies demonstrate that engaging in regular exercise and physical activity can boost the activity of antioxidant enzymes, halt neovascularization (230), and increase resistance to oxidative stress (23).

Inflammation plays a significant role in the pathophysiology of AMD. Research has shown that the AMD pathology is accompanied by the infiltration of various inflammatory cells, primarily microglia and macrophages. Inflammatory responses, mediated by the release of cytokines such as TNF-α and Interleukins, activate signaling pathways including NF-κB and MAPK. This process not only damages RPE and photoreceptor cells but also enhances the transcription and translation of the VEGF gene, inducing retinal neovascularization (231). Laser-induced choroidal neovascularization in CNV mice was restrained by experimental depletion of macrophages (232). Studies indicate that exercise and physical activity play a significant role in inhibiting the inflammatory response through down-regulating the expression of inflammatory factors and adhesion molecules, promoting the release of the anti-inflammatory adipokine lipocalin, decreasing macrophage activation and aggregation, and inhibiting microglial cell expression (218, 233) (see Table 3).

## 4 Conclusion and perspectives

**Figure 2:**
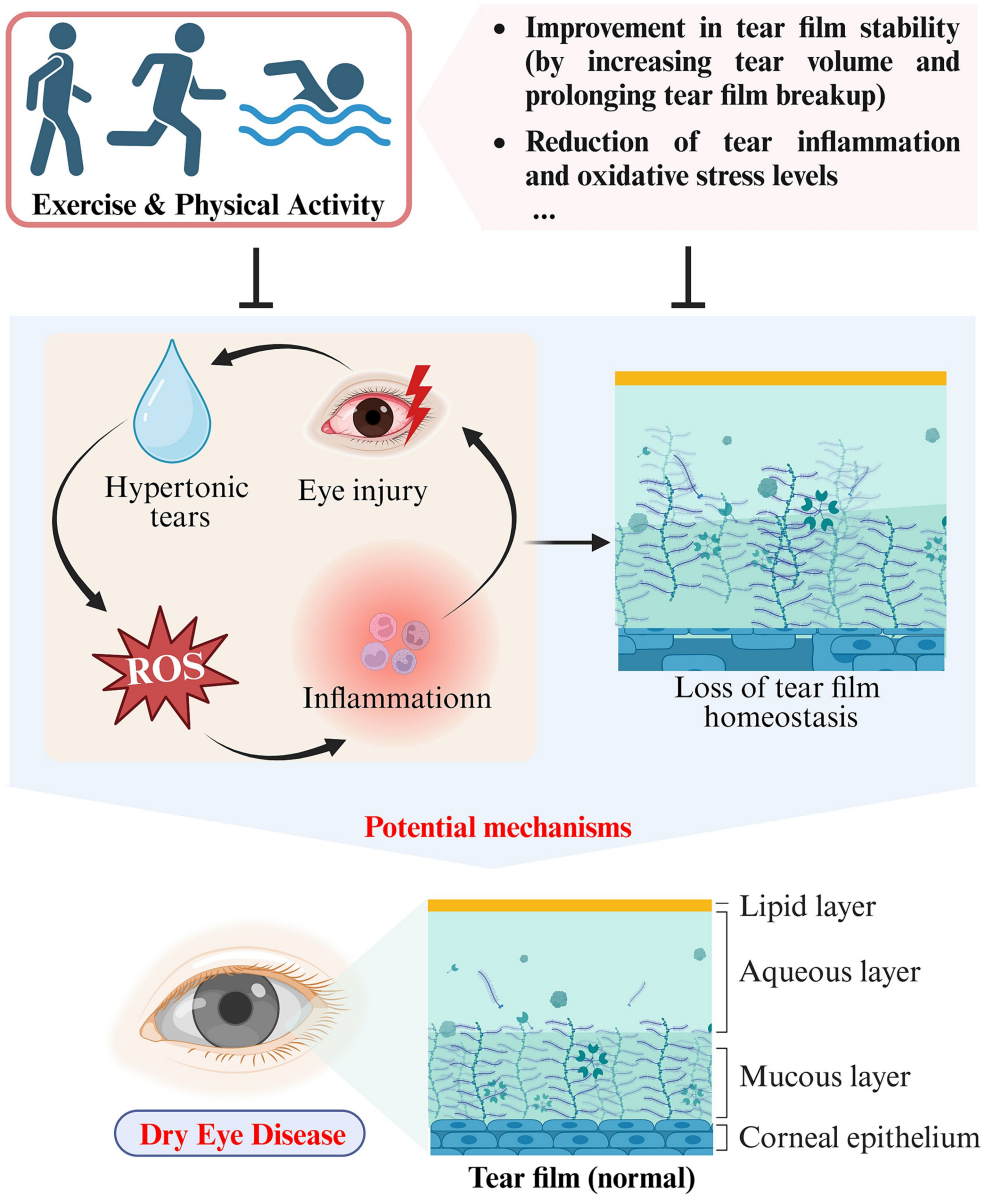
Schematic representation of the impact and potential mechanisms of exercise and physical activity on DED (created with Biorender.com).

**Figure 3:**
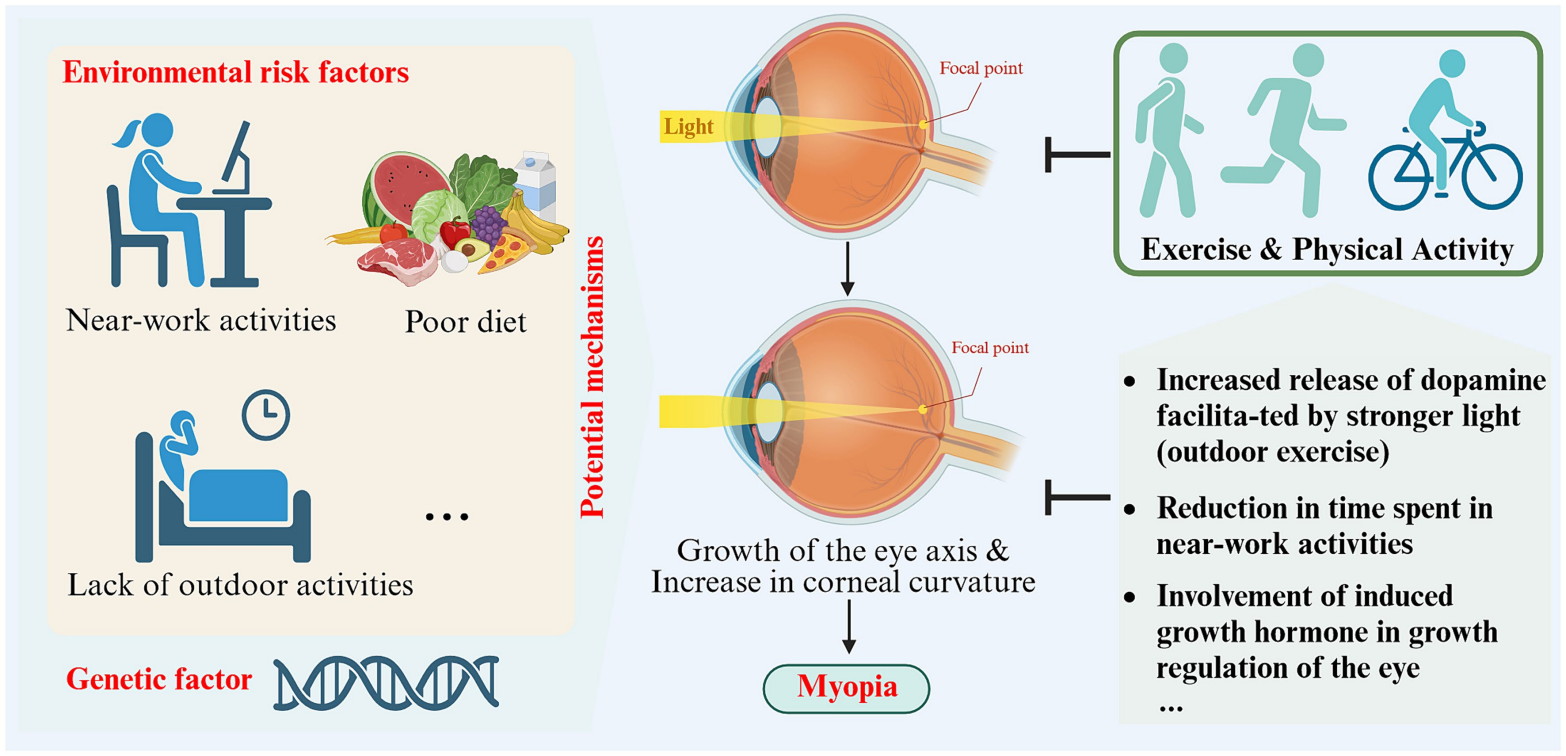
Schematic representation of the impact and potential mechanisms of exercise and physical activity on myopia (created with Biorender.com).

**Figure 4:**
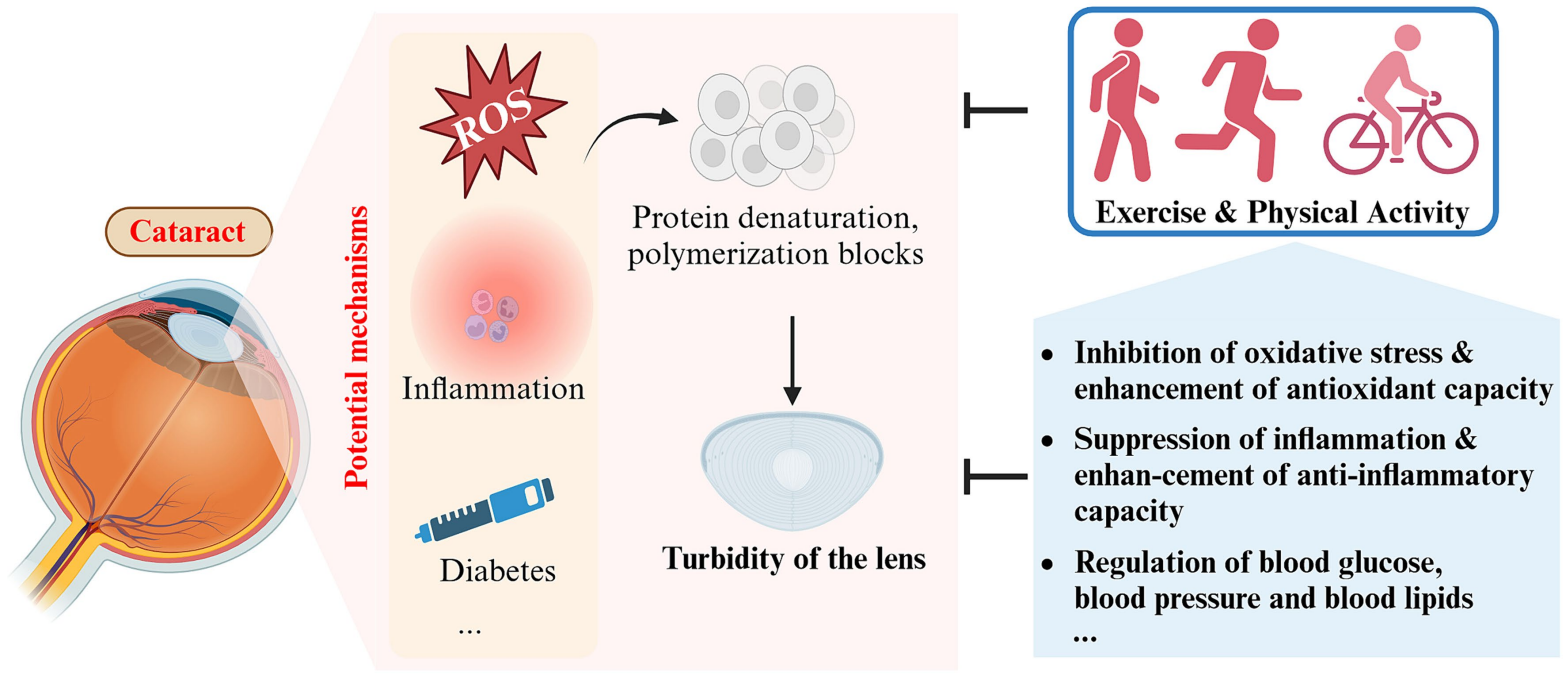
Schematic representation of the impact and potential mechanisms of exercise and physical activity on cataract (created with Biorender.com).

**Figure 5:**
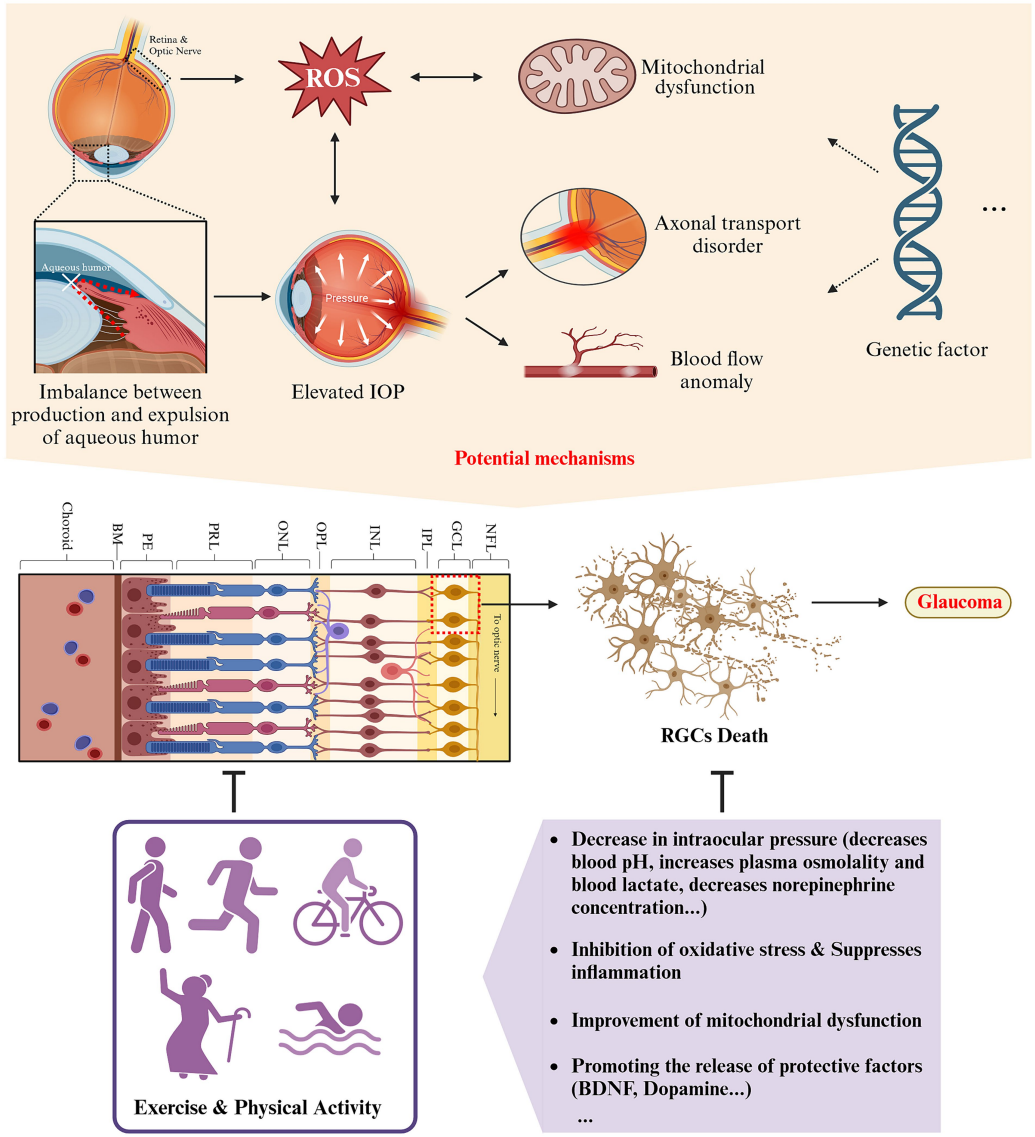
Schematic representation of the impact and potential mechanisms of exercise and physical activity on glaucoma (created with Biorender.com).

**Figure 6:**
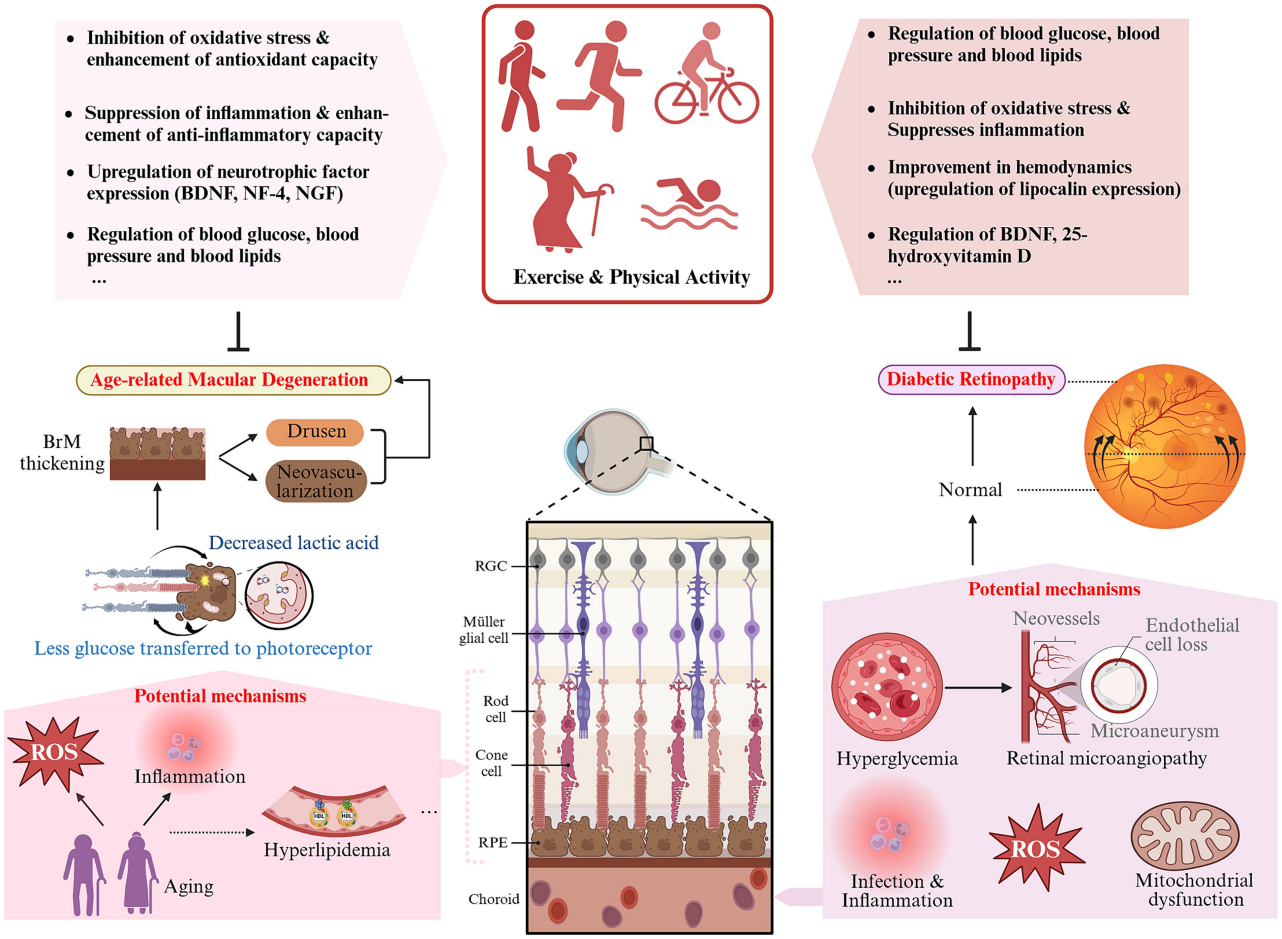
Schematic representation of the impact and potential mechanisms of exercise and physical activity on DR and AMD (created with Biorender.com).

This review synthesizes the current knowledge on the impact and potential mechanisms of exercise and physical activity on common ocular diseases such as DED, cataracts, myopia, glaucoma, DR, and AMD, as depicted in Figures 2–6. It provides a relatively comprehensive elucidation of the relationship between physical activities and eye health. According to existing research, it can be inferred that for individuals suffering from DED, myopia, cataracts, glaucoma, DR, and AMD, or those at high risk, when physical activity is appropriately tailored to their conditions, long-term, regular exercise of moderate to vigorous intensity can help delay the onset and progression of these diseases or alleviate their symptoms. This offers a promising avenue for the prevention and treatment strategies of a variety of ocular diseases. Aligning with the Global recommendations on physical activity for health and United States Health and Human Services physical activity guidelines for adults (25, 26), combined with the findings of this review, we propose that for early to mid-stage patients or at-risk groups of these ocular diseases, long-term adherence to moderate-intensity physical activities (≥150 min per week) like running, walking, and cycling in suitable environments (considering temperature, humidity, light intensity, etc.) could be instrumental in preventing or delaying the progression of these common eye conditions.

The impact of exercise and physical activity on other eye disorders has also been thoroughly researched. For instance, studies on retinitis pigmentosa (RP) have discovered that patients maintaining higher levels of physical activity exhibit superior overall visual function, color vision, and peripheral vision compared to their less active counterparts, despite the limited sample size and reliance on subjective questionnaire surveys. These findings necessitate further validation in future research (234, 235). Additionally, the benefits of exercise and physical activity on retinal microvasculature, including reductions in blood pressure and glucose levels, decreased plasma viscosity, and enhanced antioxidative capacity of red blood cells (236–239), suggest potential improvements in the development and progression of retinal vein occlusion (RVO), a condition significantly influenced by high blood sugar, hypertension, arteriolar narrowing, or occlusions (240). Moreover, the positive effects of exercise and physical activity on autoimmune diseases, such as rheumatoid arthritis, may extend to uveitis, a common extra-articular manifestation of rheumatoid arthritis (4, 241, 242). In the regulatory mechanisms, a novel myokine, irisin, induced by exercise and secreted by skeletal muscles, has been shown to be downregulated in the aqueous humor of patients with RVO and AMD, as well as in the retinas of mice with oxygen-induced retinopathy, suggesting it may be a marker of retinal damage (137, 243). Current research indicates that irisin can exert anti-inflammatory, antioxidative, and anti-apoptotic neuroprotective effects through several signaling pathways, including Akt and ERK1/2 (244), integrin αVβ5/AMPK (245), and ROS-NLRP3 (246), among others. Further investigation into the impact of this molecule on ocular diseases may enrich the understanding of exercise’s protective mechanisms and potentially offer a new therapeutic strategy for neuroprotection of the visual system.

It’s noteworthy that in epidemiological studies included in this review, the form and intensity of exercise were adapted to the subjects. Under this premise, the positive impact of exercise or physical activity on dry eye syndrome, cataracts, glaucoma, diabetic retinopathy, and other ocular diseases has been validated. However, it is undeniable that in certain specific situations, exercise and physical activity should be reduced or even temporarily prohibited. These include: (1) late stages or acute episodes of vision-impacting ocular diseases, such as cataracts, glaucoma, diabetic retinopathy, where the patient’s vision and/or visual field is severely compromised, making the positive impact of exercise and physical activity almost negligible and potentially exacerbating the condition or increasing the risk of accidental injury (111, 247, 248); (2) the recovery period following ocular surgery (e.g., cataract surgery, vitrectomy, laser treatment), where vigorous exercise should be avoided to ensure proper healing of the wound (249, 250); (3) environments that are dry, windy, or dusty may exacerbate symptoms of dry eye syndrome, and intense sunlight or dim lighting may worsen eye pressure issues in glaucoma patients (251–253); (4) patients with high myopia are at increased risk of retinal detachment and should avoid high-impact and vibration-inducing activities, such as running and lifting heavy objects (254); (5) when specific complications are present, such as diabetic retinopathy patients with concurrent macular edema, short-term high-intensity exercise should be avoided (255).

This review aims to summarize the positive effects of exercise and physical activity on common ocular diseases, but this does not imply that exercise and physical activity can directly serve as a treatment modality. Seeking medical assistance and clinical treatment is necessary and primary for any ocular disease. We hope this review serves as a catalyst to stimulate interest among researchers and medical professionals in further exploring the relationship between exercise and eye health. We look forward to future studies revealing the specific mechanisms and key targets through which exercise benefits ocular health, thereby facilitating the development of innovative strategies for treating eye diseases.

This review also has limitations. Different studies may involve participants of varying ages, genders, races, etc., and employ different forms of exercise and physical activity, leading to variability in type, intensity, frequency, and duration. The classification of exercise intensity in this review utilizes metabolic equivalents (METs), a widely accepted standard. However, it is not the sole method available; other measures such as maximum oxygen uptake (VO_2_ Max), heart rate, and heart rate reserve also have their advantages. Relying on a single measurement standard could potentially affect our interpretation and summary of the outcomes. Additionally, long-term follow-up studies included in the review may be subject to response bias, such as self-reported exercise and physical activity data potentially being influenced by respondents’ subjective biases. There are also certain limitations and gaps in research on different types of eye diseases. For example, some studies may not have accounted for confounding factors, such as genetic predispositions, environmental factors, and behavioral factors (e.g., near work and screen time usage), making it difficult to ascertain the impact of exercise on dry eye syndrome and myopia. In the case of diseases like AMD, existing research lacks unified recommendations for specific types of exercise, intensity, frequency, and duration, making it challenging to provide optimal exercise guidelines.

## Author contributions

QZ: Writing – original draft, Writing – review & editing. YJ: Writing – original draft, Writing – review & editing. CD: Writing – original draft, Writing – review & editing. JW: Writing – original draft, Writing – review & editing.
