# Corrigendum: Effects and potential mechanisms of exercise and physical activity on eye health and ocular diseases

**DOI:** 10.3389/fmed.2024.1427623

**Published:** 2024-05-16

**Authors:** Qiuxiang Zhang, Yuxian Jiang, Chaohua Deng, Junming Wang

**Affiliations:** Department of Ophthalmology, Tongji Hospital, Tongji Medical College, Huazhong University of Science and Technology, Wuhan, China

**Keywords:** exercise, physical activity, ocular diseases, oxidative stress, inflammation

In the published article, there was an error in the legends for Figures 1–6 as published. The relevant citation “created with Biorender.com” was missing. The corrected legends appear below.

Figure 1. Type, intensity and recommended amount of exercise and physical activity (created with Biorender.com).

Figure 2. Schematic representation of the impact and potential mechanisms of exercise and physical activity on DED (created with Biorender.com).

Figure 3. Schematic representation of the impact and potential mechanisms of exercise and physical activity on myopia (created with Biorender.com).

Figure 4. Schematic representation of the impact and potential mechanisms of exercise and physical activity on cataract (created with Biorender.com).

Figure 5. Schematic representation of the impact and potential mechanisms of exercise and physical activity on glaucoma (created with Biorender.com).

Figure 6. Schematic representation of the impact and potential mechanisms of exercise and physical activity on DR and AMD (created with Biorender.com).

The authors apologize for this error and state that this does not change the scientific conclusions of the article in any way. The original article has been updated.

